# Elucidation of the mechanism Underlying the promotion of ferroptosis and enhanced antitumor immunity by citrus polymethoxyflavones in CRC cells

**DOI:** 10.3389/fphar.2025.1571178

**Published:** 2025-04-11

**Authors:** Yingying Duan, Yu Wu, Jiaqi Tian, Yuqin Yin, Zhongwen Yuan, Wenting Zhu, Suyue Zhou, Chen Li, Senling Feng

**Affiliations:** ^1^ Guangdong Provincial Key Laboratory of Major Obstetric Diseases, Department of Pharmacy, Guangdong Provincial Clinical Research Center for Obstetrics and Gynecology, The Third Affiliated Hospital, Guangzhou Medical University, Guangzhou, China; ^2^ School of Pharmaceutical Sciences, Guangzhou Medical University, Guangzhou, China; ^3^ Department of Pharmacy, The First Affiliated Hospital of Guangxi Medical University, Nanning, China

**Keywords:** colon cancer, citrus polymethoxyflavones, ferroptosis, PD-L1, antitumor immunity

## Abstract

**Background:**

Colon cancer is a prevalent condition with a high mortality rate on a global scale. Research has indicated that Citrus polymethoxyflavones (PMFs), a class of flavonoids found in Citrus, possess the potential to demonstrate anti-tumor efficacy. Ferroptosis a form of cell death that is dependent on iron accumulation and lipid peroxidation. Immunotherapy is one of the most commonly used anti-tumor modalities in a clinical setting. Consequently, studies on the pharmacodynamic mechanism of Citrus to determine whether it can modulate tumor immunity through ferroptosis provide new ideas for the clinical treatment of colon cancer.

**Purpose:**

The objective of this study is to ascertain whether Citrus inhibits *PD-L1* through ferroptosis and promotes tumor immunity among patients with colon cancer.

**Methods:**

The inhibitory effect of PMFs on colon cancer was proved by *in vitro* experiment and *in vivo* model. In addition, the occurrence of ferroptosis was detected by measuring key ferroptosis indicators. Bioinformatics analysis was then performed to identify the crossover genes for Citrus polymethoxylflavonoids, colon cancer, and ferroptosis. Finally, key genes were identified by immunocorrelation analysis including WB, Q-PCR and flow cytometry. These experiments were designed to reveal the potential mechanisms of PMFs on ferroptosis and anti-tumor immunity.

**Results:**

*In vitro* cell proliferation experiment and the growth of transplanted tumor mice showed that PMFs had inhibitory effect on colon cancer. In addition, the change of ferroptosis index showed that PMFs promoted the occurrence of ferroptosis, followed by Q-PCR and WB detection of *NOX4* and *TIMP1*, the key genes screened by bioinformatics, found that PMFs inhibited *PD-L1* by down-regulating *TIMP1*, thus affecting colon cancer. Flow cytometry showed that CD4^+^ T expression increased and CD8^+^ T cell expression decreased after treatment, suggesting that anti-tumor immunity was activated.

**Conclusion:**

It is conceivable that the tumor immune microenvironment may be subject to regulation during the inhibition of colon cancer through ferroptosis in PMFs. The ferroptosis-related gene *TIMP1* has been observed to regulate *PD-L1*, thereby promoting anti-tumor immunity in colon cancer. However, further investigation is required to ascertain the underlyingprecise mechanisms.

## Graphical Abstract

**Figure:**
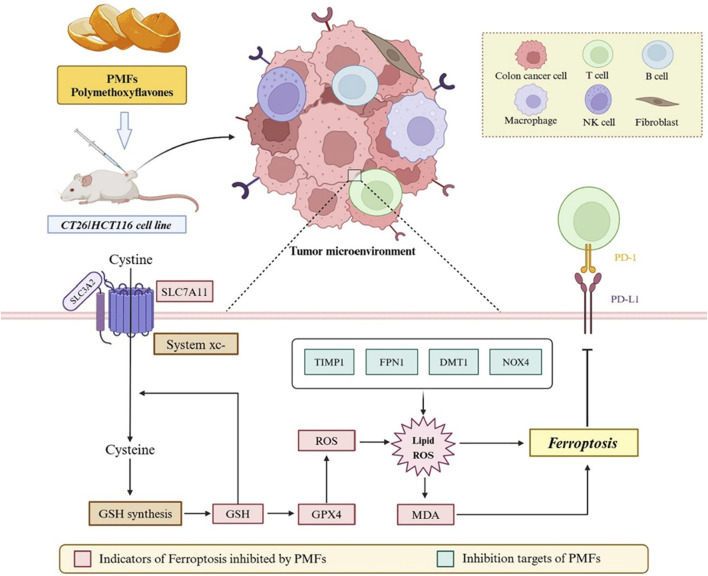
Validation of the effect of Citrus polymethoxyflavonoids on the ferroptosis pathway in relation to the colon cancer tumor microenvironment by in vitro cellular experiments and in vivo xenograft tumor model.

## 1 Introduction

Colon cancer (CC) is a prevalent and malignant neoplasm that has a significant global impact. The most recent statistics indicate that the global incidence of colon cancer is the second highest among women and the third highest among men (Sung et al., 2021). A review of related studies indicates that from the 2000s to 2023, there has been a rapid shift in the incidence of colon cancer in the United States population towards younger and older age groups (Siegel et al., 2023). Conversely, the incidence and mortality rates of colon cancer in China account for 22.6% and 33.4% of the global total, respectively, which are higher than the global average (Lv et al., 2022). Owing to the substantial population base, the incidence and mortality rates are the highest on a global scale. In light of the latest revisions to clinical colon cancer guidelines, it is now recommended that screening for the disease be initiated at the age of 45, years to address the increasingly younger age group of patients affected by the disease (Fabregas et al., 2022). Consequently, treatment and prognosis of colon cancer have become a significant concern within the medical community.

Historically, chemotherapeutic agents constituted the primary conventional drugs employed in the treatment of colon cancer. However, recent advancements in research have elucidated the roles of chronic inflammation, tumor-induced inflammation, the TME (tumor microenvironment), and certain adaptive immune cells in the development of CC (Dmitrieva-Posocco et al., 2019; Greten and Grivennikov, 2019; Ziegler et al., 2018; Goldszmid et al., 2015). In recent years, immunotherapy has emerged as a more effective novel therapeutic option for cancer treatment. This innovative therapeutic modality is predicated on the intricate interplay between cancer cells and the immune system to fosterelicit anti-tumor immunity, and it has gained considerable traction, particularly in the applicationcontext of ICI (Immune Checkpoint Inhibitor Therapy), which has emonstrated efficacy in cases of colon cancer with defective mismatch repair and high microsatellite instability. The mechanism by which ICI exerts its effects involves the inhibition of the negative regulation of receptors on T cells, including but not limited to *CTLA-4* (cytotoxic T-lymphocyte antigen-4) and *PD-1* (programmed death protein 1). The consequence of this inhibition is an enhancement of the anti-tumor immune response (Lichtenstern et al., 2020; Sharma et al., 2021; Topalian et al., 2015; Wei et al., 2018). This therapy has notable advantages over traditional cancer treatments. While response rates are typically below 20%, patients who do respond demonstrate more prolonged responses (Zugazagoitia et al., 2016; Franke et al., 2019). Consequently, it is imperative to investigate the potential target mechanisms of ICI in colon cancer.

Ferroptosis was initially identified as a unique and distinct form of RCD (regulated cell death), separate from necrosis and autophagy observed in oncogenic degenerative RAS mutant cancer cells. The targeting of ferroptosis is considered a promising approach for the treatment of numerous cancers, including CRC (Colorectal Cancer) (Jiang et al., 2021). It is well established that ferroptosis is characterized by the accumulation of iron and lipid peroxidation, which leads to the formation of lipid peroxides. ROS (Reactive oxygen species) play an essential role in coordinating crosstalk between various immune cells and creating a toxic environment for cancer cells. At the same time, they can prevent normal cells from being peroxidized (Wang et al., 2021; Huang et al., 2023). In addition, RSL3 has been shown to induce ferroptosis and elevate the levels of ROS and cellular destabilizing iron pools in a time - and dose - dependent manner in three CRC cell lines (Sui et al., 2018). Furthermore, it has been demonstrated that the combination of cisplatin and erastin synergistically induces ferroptosis, resulting in the significant inhibition of CRC cell line growth. This evidence suggests that ferroptosis represents an alternative mechanism of CRC cell death (Guo et al., 2018).

It has been demonstrated that the principal constituents of PMFs, in Citrus, namely, nobiletin and tangeretin, can impede tumor proliferation in a range of cancerous tissues *in vitro* (Raza et al., 2020; Kisacam, 2023; Chen et al., 2015). It is particularly noteworthy that these two natural components, when administered alone or in conjunction with the clinical anticancer drug 5-FU (5-fluorouracil), have been observed to exert a profound inhibitory effect on tumor growth (orrado et al., 2023; Dey et al., 2020). Given the bioinformatics technology screening data, further analysis of the core genes and related signaling pathways is necessary to explore how Citrus regulates colon cancer tumor immunity through ferroptosis. This analysis may provide new ideas for solving the difficult problems faced by colon cancer treatment. The idea of the line is shown below.

## 2 Materials and methods

### 2.1 Instruments

Thermo Fisher Scientific’s CO_2_ Incubator 311 was used for cell culturing. An Inverted fluorescence microscope (Ts2, Nikon, Japan) and an Orthogonal fluorescence microscope (Nikon ECLIPSE Ti2) was used for detailed cellular imaging. A Full-featured gel imaging system (Gel DocEZ, Bio-Rad, United States) was utilized for gel documentation, and a Flow cytometry system (Attune NxT V4) was employed for detailed cell analysis.

### 2.2 Reagents

Solarbio (Beijing, China) supplied MTT (M8180), PBS (P1020), Calcein-AM/EthD-I kit (CA1631), GSH kit (BC1175), MDA kit (BC0025), Ferrous ion kit (BC5415), and ROS kit (CA1410). DMSO (20230716) was purchased from Sinopharm Chemical Reagent Co., Ltd. PMFs were procured from Guizhou HYT-BlueF Co., Ltd., China. 4% paraformaldehyde solution (P1110) and 0.1% crystal violet staining solution (G1062) purchased from Solarbio were used for cell fixation and staining. The SP Rabbit & Mouse HRP Kit (DAB) from CWBIO (09423) was used for immunohistochemistry.

### 2.3 Animals

Male BALB/c mice aged between 6 and 8 weeks were procured from Zhuhai BesTest Bio-Tech Co., Ltd. (Zhuhai, China). The mice were housed in SPF environments with unrestricted access to food and water. All animal studies were conducted according to the legal regulations and national guidelines for the care and maintenance of laboratory animals.

### 2.4 Cell culture

Cellular experiments were conducted using two colon cancer cell lines to ensure the reproducibility and relevance of the findings across different genetic backgrounds. CT26, a mouse-derived colon cancer cell line, and HCT116, a human-derived colon cancer cell line, were cultured in controlled conditions using the CO_2_ Incubator 311 to maintain optimal growth environments.

### 2.5 *In vitro* efficacy and mechanism

#### 2.5.1 MTT assay protocol

Cells were seeded at a density of 5,000 cells per well in 96-well plates and incubated overnight at 37°C in a 5% CO_2_ atmosphere to allow for adherence. Post-adherence, cells were treated with predetermined concentrations of the Citrus compounds, Following a 48 h treatment period, 100 μL of MTT working solution was added to each well, and the plates were further incubated for 4 h. The reaction was terminated by adding DMSO to dissolve the formazan crystals formed. An enzyme counter with low-speed oscillation measured the absorbance, with results recorded and analyzed to assess cell proliferation.

#### 2.5.2 Cell cloning assay

For the cell cloning assay, 1,000 cells were seeded per well in 6-well plates and incubated overnight to promote attachment. Post-attachment, cells received Citrus compound treatments, with media changes every 3 days. After 14 days, the culture was terminated, and the medium was removed. Cells were washed thrice with PBS, fixed with 4% paraformaldehyde for 30 min, and stained with 0.1% crystal violet for 15 min. Post-staining, cells were washed to remove background staining, dried, and photographed for colony formation analysis.

#### 2.5.3 Live-dead staining

Cell viability and mortality were further quantified using a live-dead staining assay, following the protocol provided in the Calcein-AM/EthD-I kit. Cells were stained according to kit instructions and visualized under an inverted fluorescence microscope. Images were captured and quantitatively analyzed using ImageJ software to determine the ratio of live to dead cells, providing a robust measure of cell survival post-treatment.

### 2.6 *In vivo* drug efficacy and mechanism

#### 2.6.1 Subcutaneous transplantation of colon cancer model

To assess the therapeutic efficacy of specific treatments in colon cancer, an *in vivo* study was conducted using BALB/c mice. This study was conducted in strict accordance with the ethical guidelines established by the Ethics Committee of the Third Affiliated Hospital of Guangzhou Medical University (Approval ID: G2024-064), ensuring adherence to the highest standards of animal welfare and ethical research practices. Following acclimatization, CT26 colon carcinoma cells were subcutaneously transplanted into the mice. Treatment initiation occurred when tumor volumes exceeded 100 mm^3^. A total of 24 mice were randomized into three distinct groups: a control group receiving Control, a group treated with the chemotherapeutic agent 5-FU, and a group receiving treatment with PMFs. The dosing concentration for the treatment groups was standardized at 30 mg/kg. The administration schedule involved dosing every 2 days, continuing until the tumor volume approached nearly 3,000 mm^3^, at which point treatment was discontinued. The mice were fasted the night before the tumor excision. After the tumor was removed, its weight was measured. Part of the tumor was fixed in tissue fixative for sectioning, while another part was stored at −80°C for subsequent experimental processing.

#### 2.6.2 Hematoxylin-eosin staining

During the experiment, the paraffin sections were immersed in xylene on two separate occasions, each for a duration of 10 min, to ensure complete paraffin removal. Subsequently, the dewaxed sections were subjected to a gradient hydration process using ethanol concentrations from highest to lowest (100%, 95%, 90%, 80%, 70%), each for 2 min, to facilitate tissue rehydration to an aqueous state. Following rehydration, the sections were stained with hematoxylin for 15 min, then rinsed under running water to achieve a blue hue. The blue-stained sections were then immersed in an eosin dye solution for 5 min to selectively color the cytoplasm and extracellular matrix. To conclude the staining protocol, the sections underwent a dehydration sequence in an ascending ethanol gradient (70%, 80%, 90%, 95%, 100%), each step lasting between 1 and 2 min, followed by two xylene treatments, each for 10 min. The prepared slides were lastly sealed with neutral gum and a coverslip.

#### 2.6.3 Immunohistochemical detection

Colon tissue samples were procured under the ethical protocols approved by the Ethics Committee of the Third Affiliated Hospital of Guangzhou Medical University (Approval No. G2024-064), with informed consent obtained from all patients involved. The specimens were obtained from the Third Hospital of Guangzhou Medical University, Guangzhou, China. Each tissue sample was subjected to a standardized preparation process beginning with baking at 65°C for 1 h. The samples were then sequentially treated with xylene (I, II), anhydrous ethanol, 95% ethanol, and 75% ethanol for specified durations to accomplish dewaxing. Antigen retrieval was performed using sodium citrate with subsequent natural cooling to room temperature. The staining procedure was conducted according to the specifications of the SP Rabbit & Mouse HRP Kit (DAB). After air drying, the tissue samples were imaged under an Orthogonal fluorescence microscope (Huang et al., 2022).

### 2.7 Ferroptosis index detection

To evaluate the occurrence of ferroptosis when drugs target tumor cells, specific biochemical markers and protein expressions associated with iron-related cell death were meticulously analyzed. The assessment was facilitated by quantitative detection of key indicators using Solarbio’s assay kits. The GSH (glutathione), MDA (malondialdehyde), and ROS kits from Solarbio were employed to measure the concentration changes of glutathione, malondialdehyde, and reactive oxygen species respectively. These biomarkers are critical in determining the oxidative state within cells and the extent of lipid peroxidation, both of which are indicative of ferroptosis.

Furthermore, the expression levels of specific proteins that play pivotal roles in the regulation of ferroptosis were examined. These included *GPX4* (Glutathione Peroxidase 4), a major antioxidant enzyme that protects cells from oxidative damage; *xCT*, the cystine/glutamate antiporter essential for maintaining intracellular glutathione levels; *DMT1* (Divalent Metal Transporter 1), involved in iron uptake; and *FPN1* (Ferroportin 1), the only known iron exporter in mammalian cells. These antibodies are all from Chengdu Zhengneng Biotechnology Co., Ltd. and are diluted at a ratio of 1:1000. The expression of these proteins before and after drug treatment was observed using Western blot analysis to confirm the induction of ferroptosis as a mechanism of action by which the drugs exerted their anti-tumor effects.

### 2.8 Bioinformatics screening of key genes

#### 2.8.1 Acquisition of differential genes

A dataset comprising samples from normal individuals and colon cancer patients was procured from the GEO (Gene Expression Omnibus). This dataset serves as the foundation for conducting comprehensive transcriptomic analyses to explore the molecular underpinnings of colon cancer.

To identify genes that are differentially expressed between colon cancer samples and controls, a rigorous differential analysis was conducted using the R package limma (version 3.40.6). This bioinformatics tool is renowned for its ability to efficiently handle statistical analysis of gene expression data and is particularly adept at identifying DEGs (differentially expressed genes) with high precision.

Following the differential analysis, volcano plots were generated to visually represent the DEGs. These plots are instrumental in distinguishing genes with significant alterations in expression levels, facilitating a straightforward visualization of both the magnitude of expression changes and the statistical significance associated with these changes. The use of volcano plots aids in the rapid identification of genes that may play pivotal roles in the pathogenesis of colon cancer or could potentially serve as biomarkers or therapeutic targets.

#### 2.8.2 WGCNA weighted co-expression network

To delineate the complex molecular interactions within colon cancer, a WGCNA (weighted gene co-expression network analysis) was conducted. Initial steps involved the preprocessing of the dataset to remove batch effects, followed by the selection of appropriate soft thresholds essential for constructing robust gene co-expression networks. Subsequent stages included the identification of gene sets, delineation of co-expression modules, and exploration of the relationships between these modules and phenotypic traits. Modules demonstrating heightened relevance to colon cancer phenotypes were meticulously selected for further analysis.

#### 2.8.3 Disease genes from external databases

To broaden the scope of potential gene targets, searches were conducted in several genetic and disease databases including GeneCards, OMIM (Online Mendelian Inheritance in Man), and the TTD (Therapeutic Target Database). These searches, guided by the keyword “colon cancer”, furnished additional relevant gene targets, enriching the pool of candidates for subsequent analyses.

#### 2.8.4 Identification of targets in citrus polymethoxyflavones

Investigation into the bioactive components of Citrus polymethoxyflavones required querying databases such as TCMSP (Traditional Chinese Medicine Systems Pharmacology), Swiss TargetPrediction, and STITCH version 5.0. Following the identification of target genes, detailed genetic profiles were acquired from the Uniprot database, providing comprehensive insights into the molecular underpinnings influenced by these components.

#### 2.8.5 Exploration of ferroptosis-related genes

To pinpoint genes involved in the ferroptosis process within colon cancer lesions, data was retrieved from the ferroptosis Database (http://www.zhounan.org/ferrdb). An intersectional analysis was then performed among the genes associated with colon cancer, Citrus polymethoxyflavones, and those implicated in ferroptosis, to identify genes of joint relevance.

#### 2.8.6 Clinical survival analysis and functional enrichment analysis

The intersected genes identified through the aforementioned methodologies were subjected to functional enrichment analysis using the DAVID database (DAVID: Functional Annotation Result Summary, ncifcrf.gov), focusing on KEGG pathways and GO terms. This analysis aimed to elucidate potential pathway mechanisms implicated by the core genes. Additionally, clinical survival analysis was applied to these genes to assess their prognostic value, with significant genes determined by a P value threshold of less than 0.05.

### 2.9 Mechanistic pathway exploration of key genes

#### 2.9.1 Western blot assay

To elucidate the protein expression dynamics within colon cancer cells, a precise Western blot assay was employed. Initially, cells were lysed to extract total protein, and the concentration of these proteins was accurately quantified. Protein samples were then combined with sampling buffer and exposed to heat to denature the proteins, preparing them for separation via SDS-PAGE gel electrophoresis based on molecular weight. Following electrophoresis, proteins were transferred onto a PVDF membrane using a transmembrane transfer technique. To block non-specific binding sites, the membrane was incubated with skimmed milk. Apply primary antibodies specifically targeting *NOX4* (NADPH oxidase 4), *PD-L1* (programmed death ligand 1), and *TIMP1* (tissue inhibitor of metalloproteinase-1), all from Chengdu Zhengneng Biotechnology Co., Ltd., diluted at a ratio of 1:1000, and incubate overnight at 4°C.The membrane was subsequently washed with TBST to remove unbound antibodies, followed by the addition of the appropriate secondary antibodies and incubation at room temperature for 1 h. After further washes with TBST to ensure cleanliness, protein bands were visualized using a Full-featured gel imaging system.

#### 2.9.2 Q-PCR for detection of mRNA expression

During the execution of the q-PCR (quantitative real - time polymerase chain reaction) experiment, RNA was extracted from subcutaneous xenograft tissue samples of CRC using the Trizol method. This RNA was subsequently reverse-transcribed into cDNA, following which the reverse transcriptase was inactivated to terminate the reaction. Next, primers were synthesized as per design. The reaction system was prepared according to the fluorescence detection method. Real-time fluorescence quantitative PCR analysis was then conducted to determine the difference in Ct values between the target gene and the reference gene. The relative expression changes of the target gene among samples were quantified using the (2^-^ΔΔCt^) method.

### 2.10 Immunocorrelation analysis of key genes

#### 2.10.1 Bioinformatics immunocorrelation analysis

To dissect the impact of pivotal genes on the immune microenvironment in colon cancer, a comprehensive series of analyses were undertaken. These included immune infiltration analysis, correlation between immune infiltration and gene expression, analysis of specific immune cell types, and examination of immune checkpoint genes. The study utilized the harmonized and standardized pan-cancer dataset from TCGA (The Cancer Genome Atlas). Specifically, the TCGA Pan-Cancer (PANCAN; N = 10,535 patients; G = 60,499 genes) dataset, accessible through the UCSC Xena browser (https://xenabrowser.net/), was employed. Expression data for individual samples were meticulously extracted, focusing on primary blood-derived cancers (e.g., peripheral blood from TCGA-LAML) and primary tumor samples, along with metastatic samples from TCGA-SKCM. The expression levels were filtered to exclude values of 0, followed by a transformation using log_2_(x+0.001) to normalize gene expression profiles for subsequent correlation and immunological impact analyses.

#### 2.10.2 Immunohistochemistry of clinical specimens

Colon tissue samples were procured under the ethical protocols approved by the Ethics Committee of the Third Affiliated Hospital of Guangzhou Medical University (Approval No. 2022-076), with informed consent obtained from all patients involved. The specimens were obtained from the Third Hospital of Guangzhou Medical University, Guangzhou, China. The samples were tested by immunohistochemistry using the same method as Section 2.6.3 above.

### 2.10.3 Detection of immune factors by flow cytometry

Tumor tissues were initially subjected to enzymatic digestion using collagenase D and DNAse I to dissociate cells. The digestion process was conducted at 37°C for 45 min to ensure optimal cell yield. Following this, the reaction was halted with EDTA, facilitating the subsequent preparation of a single-cell suspension. Cells derived from the tumor tissue were resuspended in a dye buffer. Then, dilute the dead cell staining dye (Zombie R718™) to a concentration of 1:100 per 1 million cells to distinguish live cells, and incubate the mixture at room temperature in the dark for 30 min. After washing with PBS, add the Fc blocker (TruStain FcX, Clone: 93) at a concentration of 10 μg/mL per one million cells to reduce non-specific antibody binding. This was followed by another dark, room-temperature incubation for 30 min, and an additional wash with PBS.

Specific monoclonal fluorescent antibodies targeting CD45, CD3, CD4^+^ T cells, and CD8^+^ T cells were introduced to the cell suspension. Among them, CD45 (FITC, Clone: 30-F11), CD4 (APC, Clone: GK1.5), and CD8 (PE, Clone: 53-6.7) are each added at a concentration of 2.5 μg/mL per 10^^6^ cells, while CD3 (Brilliant Violet 421™, Clone: 17A2) is added at 5 μL per 10^^6^ cells. This mixture was incubated at 4°C in the dark for 30 min to allow for the binding of antibodies to their respective antigens, a crucial step for specific immune cell identification. Following the antibody incubation, cells were washed with an appropriate volume of dye buffer to remove unbound antibodies and resuspended in the same buffer. The prepared cell suspension was then subjected to flow cytometry analysis. The antibodies used above are all from BioLegend company.

### 2.11 Statistical analysis

In the evaluation of experimental outcomes across multiple trials, data are expressed as mean ± SD (standard deviation). To ensure the robustness of the findings, advanced statistical analyses were performed utilizing GraphPad Prism version 9. Student’s t-test was employed for comparisons between two independent groups, whereas one-way ANOVA (Analysis of Variance) was utilized for analyses involving more than two groups. Statistical significance was denoted by p-values, with thresholds set at *P* < 0.05, *P* < 0.01, and *P* < 0.001. These values are indicated as *, **, and ***, respectively.

## 3 Results

### 3.1 Efficacy *in vivo* and *in vitro*

#### 3.1.1 Cell proliferation and survival experiments

**FIGURE 1:**
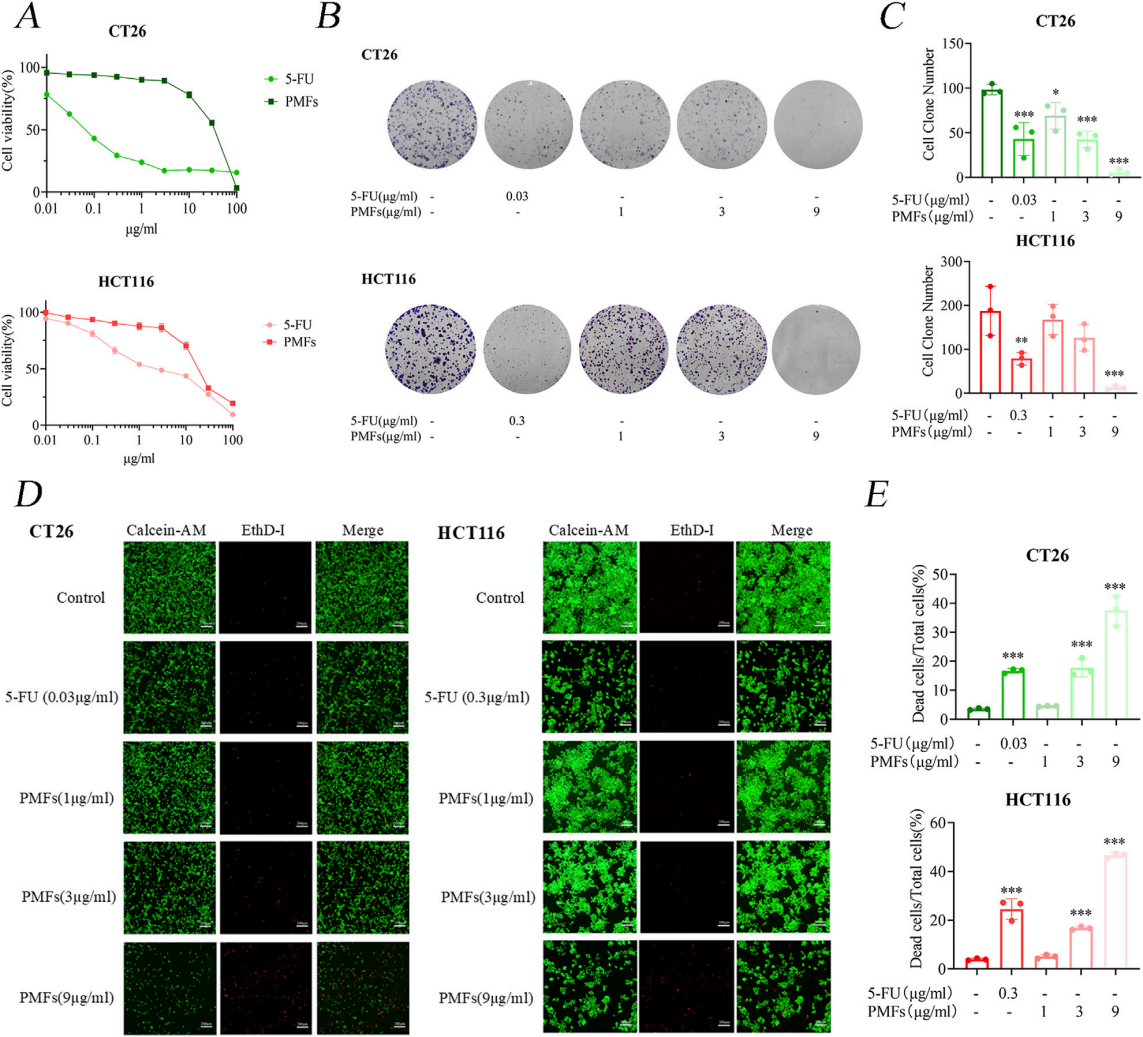
Growth inhibition and cytotoxic effects of PMFs on colon cancer cells. **(A)** CT26 and HCT116 were treated with different concentrations of PMFs and 5-FU for 48 h. Cell viability was observed as determined by MTT assay. **(B, C)** Statistical plots of cell communities and number of clones of CT26 and HCT116 plates treated with different concentrations of PMFs. **(D, E)** Fluorescence staining plots of live and dead cells and statistical plots of fluorescence proportion of dead cells as a percentage of total cells for CT26 and HCT116 treated with different concentrations of PMFs All data were obtained from three independent experiments. *P < 0.05, **P < 0.01, and ***P < 0.001.

The MTT method was set at 0, 0.01, 0.03, 0.1, 0.3, 1, 3, 10, 30, and 100 μg/mL of 5-FU (as a positive control) and PMFs after administration for 48 h. The IC_50_ was determined to be 0.06564 μg/mL and 24.997 μg/mL for CT26 cells, following the administration of 5-FU and PMFs, and the IC_50_ was established to be 2.667 μg/mL and 19.423 μg/mL for HCT116 cells, following the administration of fluorouracil and PMFs. The cell cloning and live-dead staining experiments of CT26 and HCT116 cells were set up in 5-FU and PMFs groups (Figure 1A).

Further analysis involved cloning and live/dead staining of CT26 and HCT116 cells treated with 5-FU and PMFs. The results of plate cloning indicate a significant reduction in cell proliferation in the treated group in comparison to the control group (Figures 1B, C). Furthermore, the inhibitory effect of PMFs was observed to increase in a concentration-dependent manner, emphasizing the potential dose-response relationship in the regulation of cell growth. The live and dead cell staining experiments exhibited results that were consistent with the plate cloning results, with a decrease in the proportion of green fluorescence in live cells and an increase in the proportion of red fluorescence in dead cells after administration of the treatment (Figures 1D, E).

These findings substantiate the cytotoxic capabilities of 5-FU and PMFs against colon cancer cell lines, with both agents showing considerable efficacy in reducing cell viability. The concentration-dependent inhibition exhibited by PMFs suggests its potential utility for use in tailored therapeutic applications.

#### 3.1.2 PMFs inhibit tumor proliferation in an *in vivo* mouse model

**FIGURE 2:**
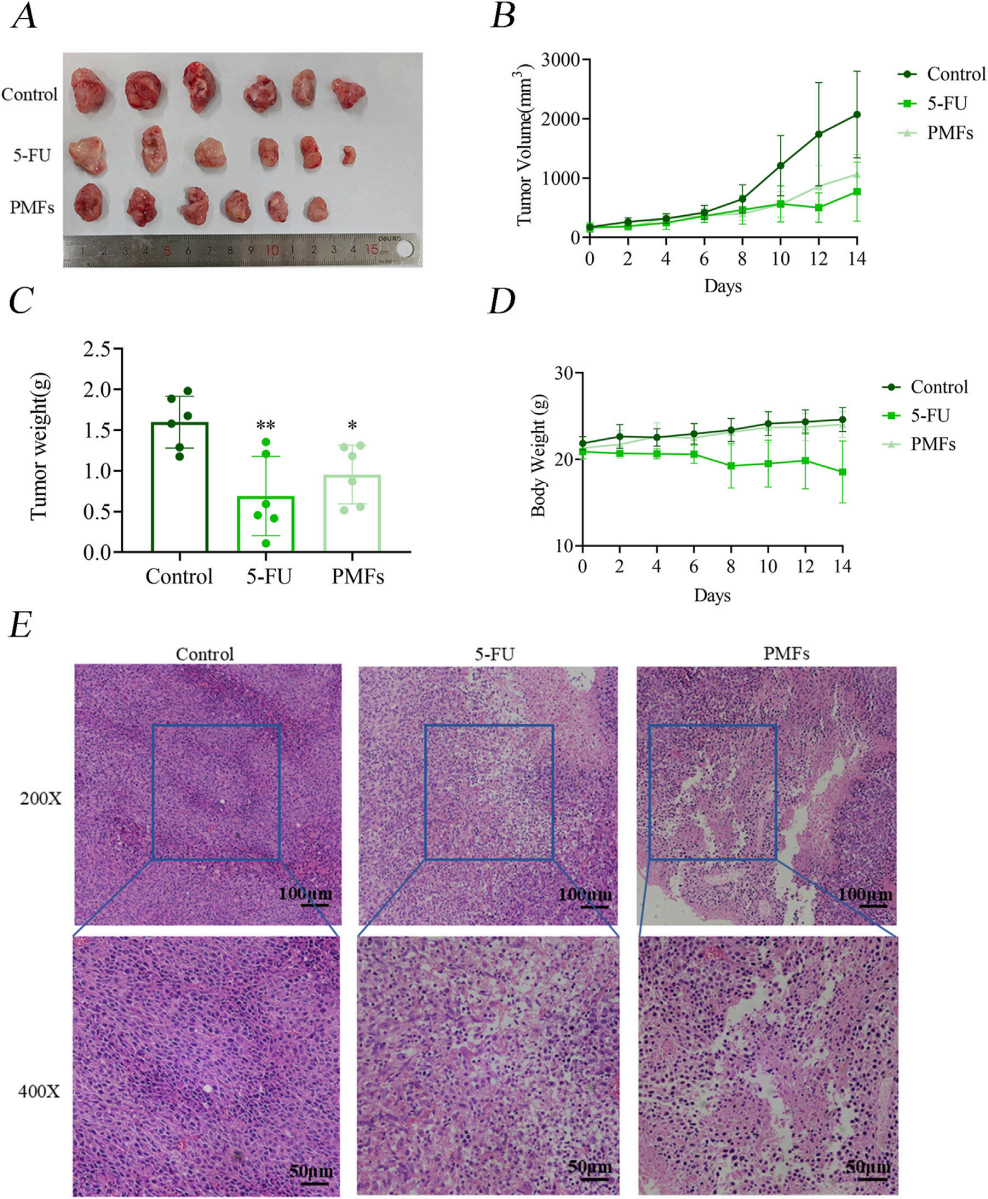
PMFs inhibits the development of xenografted tumor tissue in colon cancer. **(A–D)** Diagram of subcutaneous grafted tumour isolates in mice and statistical graphs of tumour weights in different treatment groups; line graphs of tumour volume and changes in body weight of mice during growth. **(E)** HE staining graphs of tumour tissue sections under 200X and 400x microscopy.

BALB/c mice were implanted with CT26 colon cancer cells and then treated every other day for 14 days with PMFs, 5-FU, or normal control (control group). The body weights of the mice in the 5-FU-treated group were gradually decreased, while the tumor volumes were increased. Notably, the increase in tumor volume was significantly smaller compared to the control group. mice in the PMFs group had relatively stable body weights and slower tumor growth (Figures 2A–D). HE (Histological examination under hematoxylin and eosin) staining revealed that the nuclei of the control tumor cells were typically rounded, with a high nuclear-cytoplasmic ratio, well-defined cell borders, and structural integrity. In contrast, tumor cells in both drug-treated groups exhibited structural alterations, manifesting as loose and crumpled morphology, decreased nuclear-cytoplasmic ratio, and vacuolization (Figure 2E). The above results indicate that PMFs inhibit the growth of colon cancer *in vivo* to some extent without affecting the normal physiological functions of mice.

### 3.2 Ferroptosis index detection

**FIGURE 3:**
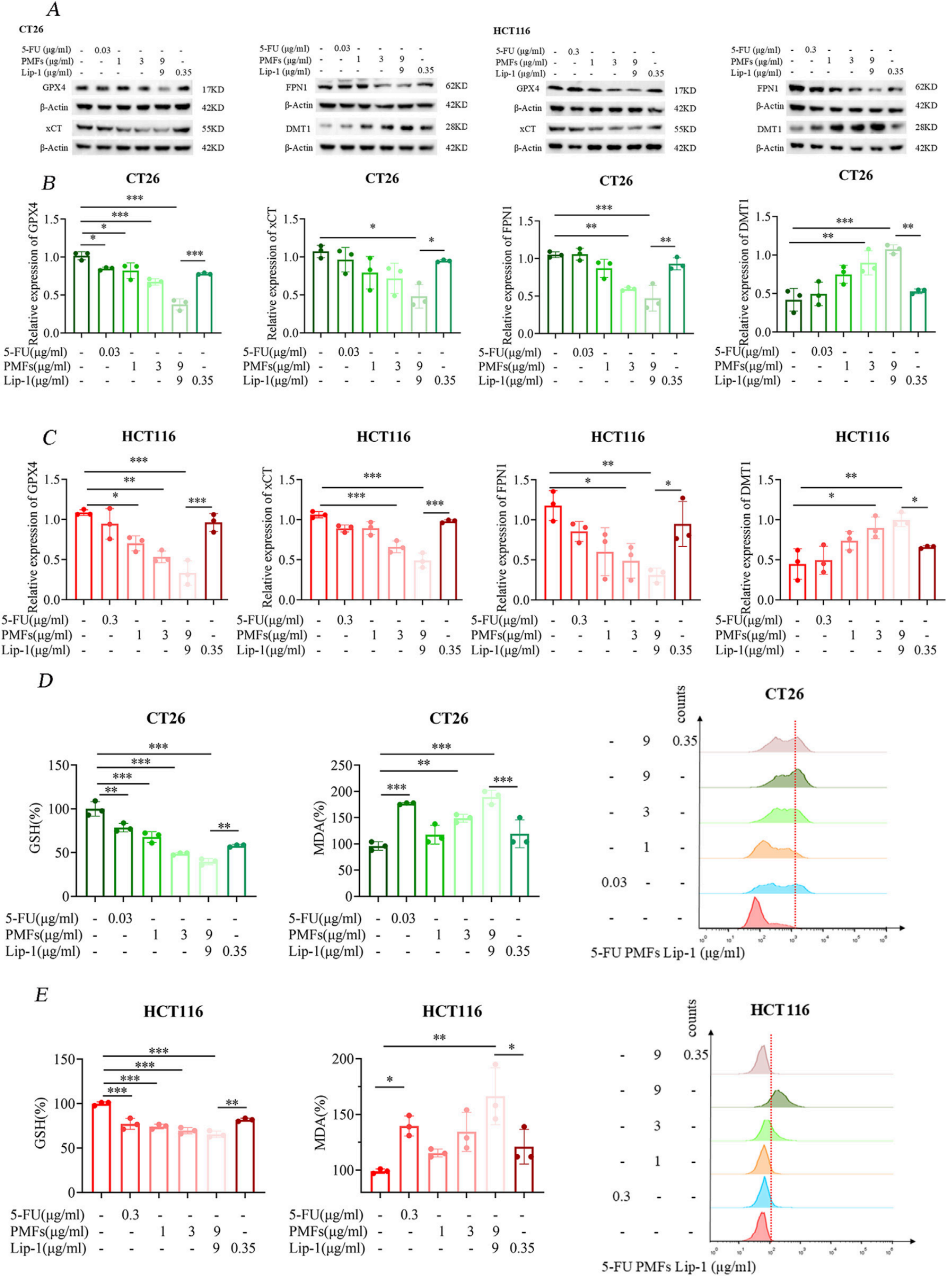
Ferroptosis mechanism of PMFs on colon cancer cells. **(A)** Changes in the bands of ferroptosis marker genes *GPX4*, *xCT*, *FPN1*, and *DMT1* after CT26 and HCT116 were treated with different concentrations of PMFs and the addition of ferroptosis inhibitor Lip-1 for 48 h. **(B, C)** Statistical plots of protein expression of the above four ferroptosis-related genes after drug treatment. **(D, E)** Changes in the expression of ferroptosis indicators GSH, MDA and ROS on CT26 and HCT116 after the same treatment with the above drugs for 48 h. *P < 0.05, **P < 0.01, and ***P < 0.001.

Utilizing Western blot analysis, we examined the expression levels of key ferroptosis-related proteins including *GPX4*, *xCT*, *FPN1*, and *DMT1* in colon cancer cells following treatment with PMFs. A significant decrease in *GPX4* and *xCT* expression was observed following PMFs administration, which was concomitant with a reduction in glutathione synthesis. Conversely, there was a decrease in *FPN1*, which is involved in the exportation of iron ions from cells, alongside an increased increment in *DMT1* (Figure 3A). These findings, were further corroborated by the application of a ferroptosis inhibitor, Lip-1, which appeared to mitigate these protein expression changes, thus suggesting a ferroptosis-driven mechanism (Figures 3B, C). Subsequent biochemical assays conducted 48 h after the commencement of treatment revealed a decline in GSH levels, accompanied by an increase in MDA levels. This finding suggests that the antioxidant defense system of the tumor cells was compromised, leading to lipid peroxidation. Enhancement of lipid peroxidation was observed. Furthermore, the analysis of PMFs-treated cells using flow cytometry demonstrated elevated levels of ROS in comparison to the control group, and that treatment with the ferroptosis inhibitor Lip-1 inhibited these responses (Figures 3D, E).

### 3.3 Bioinformatics screening of key genes

#### 3.3.1 Acquisition of differential genes

**FIGURE 4:**
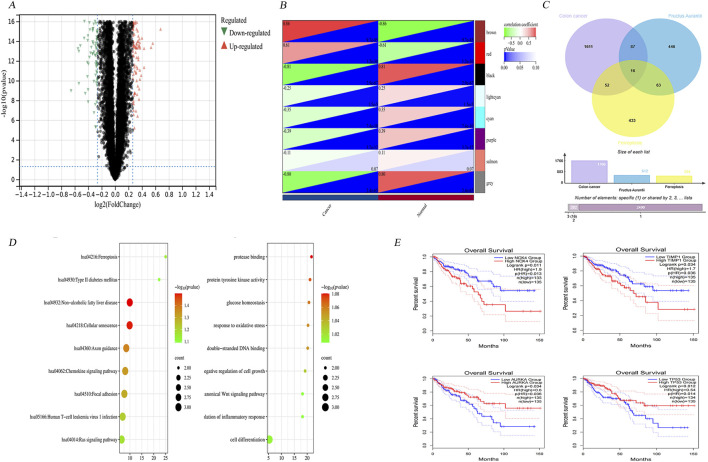
Bioinformatics screening of key genes. **(A)** Differential gene volcano map of 4 colon cancer datasets GSE146587, GSE127069,GSE44076 and GSE60697 after normalisation. **(B)** Modular gene heatmaps of the above 4 colon cancer dataset genes screened for more differential genes by WGCNA analysis. **(C)** 16 intersected genes were obtained by taking intersections of colon cancer differential genes, Hovenia-related genes and ferroptosis database genes. **(D)** The 16 intersected genes were subjected to gene ontology (GO) enrichment analysis and Kyoto Encyclopedia of Genes and Genomes (KEGG) pathway analysis to get the corresponding biological processes and related pathways. **(E)** Clinical survival analysis screened four genes with significant differences in survival prognosis as *NOX4*, *AURKA*, *TIMP1*, and *TP53*.

To identify genes exhibiting differential expression, four datasets were obtained from the GEO (https://www.ncbi.nlm.nih.gov/geo/). These datasets included samples from cancerous and non-cancerous tissues. The datasets obtained were GSE146587, GSE127069, GSE44076, and GSE60697. The GSE146587 and GSE127069 datasets comprise gene expression profiles of six healthy and six tumor patient samples, respectively. The GSE60697 dataset encompasses 20 colon cancer samples, while the GSE44076 dataset includes 148 healthy tissue samples and 98 tumor samples. The gene expression profile files of the aforementioned datasets were uploaded in TXT format to Sangbox (http: //www.sangerbox.com/tool) and subsequently merged using the R package InSilicoMerging (Shen et al., 2022; Taminau et al., 2012). Furthermore, a batch effect was observed between the four datasets due to the significant discrepancy in sample distribution. To address this, Johnson et al.'s method was employed to remove the batch effect, and the results are evident in the box-and-line plots and densitograms, confirming uniformity in data distribution post-correction (Johnson et al., 2007). As demonstrated by the UMAP plots in the Supplementary Figure S1, the distribution of the new gene expression profiling data exhibited a convergence towards a uniform distribution, with a comparable median, mean, and variance. Following the de-batching process, a clear tendency for sample clustering was observed. The combined de-batched gene expression matrices were analyzed for differences using the R package Limma (version 3.40.6) (Ritchie et al., 2015). Following this analysis 612 upregulated and 741 downregulated genes were identified as the result of screening the differential genes based on the parametric criteria of FDR <0.05 and log_2_FC > 1.2. The differential gene volcano maps were then drawn (Figure 4A).

#### 3.3.2 WGCNA weighted Co-expression network

Firstly, the MAD (Median Absolute Deviation) of the expression profiles for each gene was calculated to construct a robust co-expression network that accurately reflects the transcriptomic structure of CRC. The top 50% of genes displaying the smallest MAD values, were then removed, ensuring the focus remained on genes exhibiting significant expression variability, which are often more biologically informative. The subsequent application of the goodSamplesGenes method from the R package WGCNA facilitated the exclusion of outliers and aberrant samples, thereby refining the dataset for network construction. A scale-free network was then constructed to delineate the complex patterns of gene co-expression within the dataset. A sample clustering diagram, which provides a visual representation of the relationships between samples, can be found in Supplementary Figure S2.

Utilizing WGCNA, we further dissected the network by setting a precise cut line in the module tree diagram, which allowed for the merging of closely related modules. Modules exhibiting a distance less than 0.25 were combined, thereby optimizing the modular structure for subsequent analyses. This process culminated in the identification of 25 distinct co-expression modules, with gray modules representing genes that could not be categorized into any specific module. The correlation heatmap between these modules and clinical phenotypes highlighted the brown module as having the highest correlation with the clinical features of colon cancer (Figure 4B). Following the methodology outlined by Tang et al. (2018), hub genes within this module were identified by calculating both the GS (gene significance) and the MM (module membership). These metrics denote the correlation of genes with the clinical traits and the eigengenes of the modules, respectively.

The analytical rigor led to the identification of 550 genes characterized by high connectivity within clinically relevant modules, deemed hub genes. The selection of these genes was made on the basis of stringent cutoffs (|MM| > 0.8 and |GS| > 0.1), ensuring that only genes with strong associations to both the module eigengenes and clinical characteristics were included.

#### 3.3.3 Comprehensive disease gene integration

The genes identified in each of the three databases were integrated with the differential genes retrieved from the GEO repository and the modular genes extracted from the WGCNA analysis. Following the removal of duplicates, a total of 1,766 disease genes were identified.

#### 3.3.4 Acquisition of gene targets in citrus polymethoxyflavones

In parallel, an extensive search was carried out using the TCMSP (Traditional Chinese Medicine Systems Pharmacology) database to identify active components of Citrus polymethoxyflavones, guided by stringent selection criteria (OB (Oral Bioavailability) ≥30% and Drug Likeness (DL) ≥0.18). This was complemented by explorations in SwissTargetPrediction and STITCH 5.0 to ascertain corresponding gene targets for these active ingredients. By collating target data from these platforms into Uniprot, a total of 723 drug-target genes were identified, offering a substantial foundation for subsequent pharmacological investigations.

#### 3.3.5 Ferroptosis database genes

A total of 844 genes associated with ferroptosis were downloaded from the FerrDb database, comprising driver genes (369), marker genes (11), suppressor genes (348), and unclassified genes (116).

These genes were then cross-referenced with the screened Citrus- CRC genes and related genes, resulting in the identification of 16 genes (Figure 4C). These include *HMOX1*, *AURKA*, *MMP13*, *NOX4*, *PIK3CA*, and *TP53*. The remaining genes, *NRAS*, *EGFR*, *ABCC1*, *PPARG*, *TIMP1*, *AKR1C1*, *SRC*, *CA9*, *AHCY*, and *ADIPOQ*, will be subjected to further analysis in the subsequent section to identify the key genes. This will entail an examination of functional enrichment and clinical survival.

#### 3.3.6 Clinical survival analysis and functional enrichment analysis

The comprehensive functional enrichment analysis of 16 genes associated with ferroptosis in CRC therapies revealed pivotal biological processes and pathways (Figure 4D). The GO (Gene Ontology) enrichment analysis indicated an association with processes such as cellular hypoxia, fatty acid oxidation, regulation of fatty acid metabolism, negative regulation of cellular autophagy, and negative regulation of macrophage-derived foam cell differentiation. Conversely, KEGG enrichment was found to be linked to pathways including ROS, *PD-L1* expression, and checkpoint sites in cancer, mitochondrial autophagy, colon cancer, and ferroptosis.

To identify key genes, the intersecting genes were subjected to clinical survival analysis. Four genes were identified as having significant differences between cancerous and non-cancerous tissues via survival analysis and prognostic correlation within the 0–150 months timeframe. These genes were identified as *NOX4*, *TIMP1*, and *AURKA* (auroramitotic kinase A), and *TP53* (tumor protein P53), with their detailed clinical staging depicted in Supplementary Figure S3.

Clinical survival analyses showed that *NOX4* and *TIMP1* were associated with a poor prognosis, whereas low expression of *AURKA* and *TP53* was associated with poor prognosis (Figure 4E). The above results highlight the importance of target genes in tumour suppression and maintaining genomic stability.

### 3.4 Immunocorrelation analysis of key genes

#### 3.4.1 Analysis of core gene expression and immune infiltration

After acquiring sample data and extracting gene expression profiles for colon cancer, these profiles were annotated with GeneSymbol and further analyzed using the R package ESTIMATE (version 1.0.13) available at https://bioinformatics.mdanderson.org/public-software/estimate. This tool was employed to compute the ESTIMATE scores for each patient, providing an index reflective of the level of immune cell infiltration based on gene expression data (Yoshihara et al., 2013).

**FIGURE 5:**
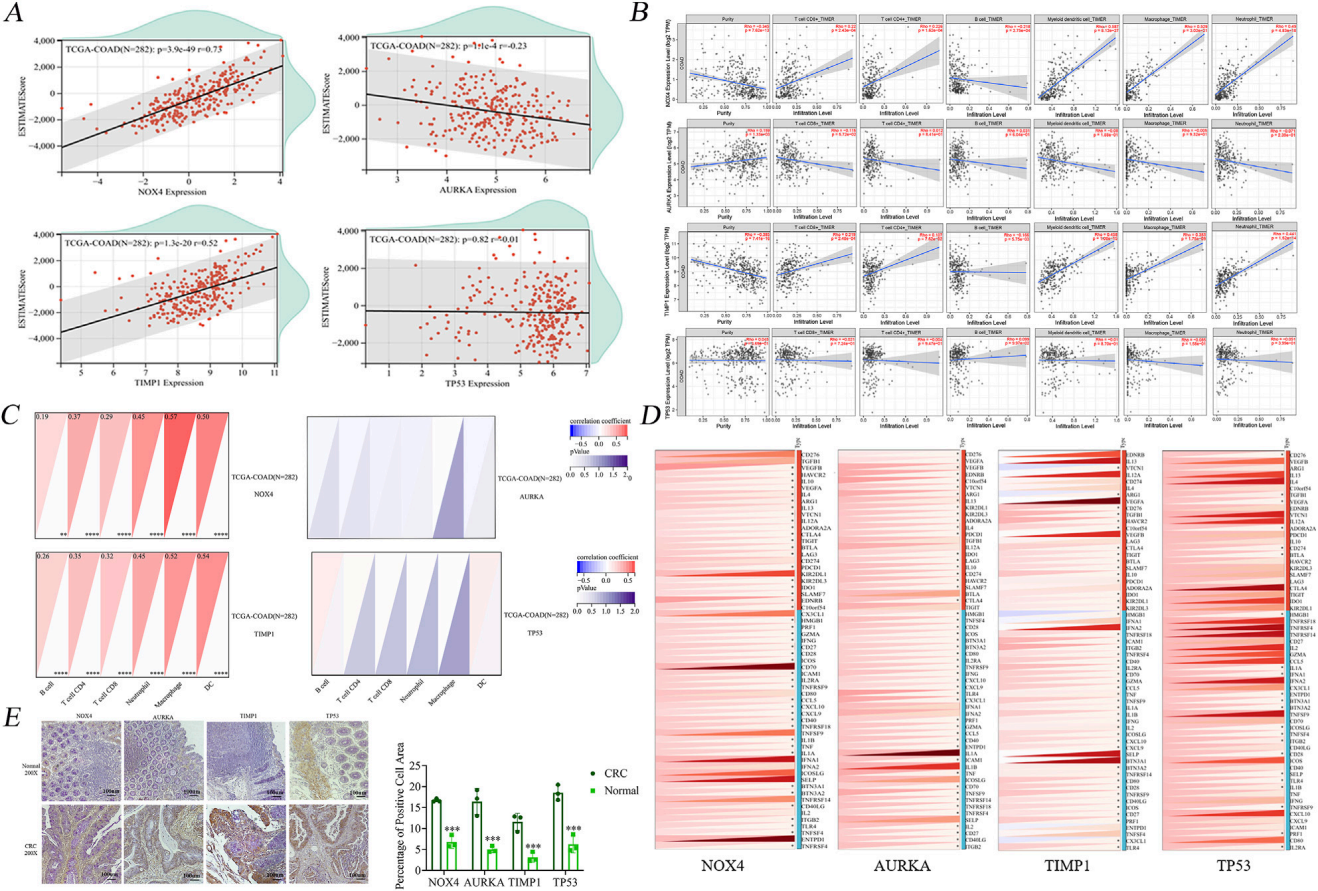
Raw letter immunological analysis of 4 key genes. **(A)** Immune infiltration analysis showed no significant correlation between *TP53* and infiltration of six immune cells. **(B)** Scatter plot of immune infiltration correlation showed no significant correlation between *AURKA* and the level of infiltration of six immune cells. **(C)** Immunocytological analysis showed no significant difference between AURKA and TP53 and the number of both *AURKA* and *TP53* and the number of six types of immune cells. **(D)** Immune checkpoint correlation analysis showed four genes were significantly associated with partial inhibitory and promotional checkpoints, respectively. **(E)** Immunohistochemical staining plots of clinically obtained human healthy tissues and colon cancer tissue specimens and statistics of the percentage of positive cells. *P < 0.05, **P < 0.01, and ***P < 0.001.

To explore the associations between gene expression and the immune microenvironment, Pearson correlation coefficients were calculated using the Corr. test function of the R package psych (version 2.1.6). This analysis facilitated the identification of significant correlation between gene expressions with immune infiltration scores. As depicted in Figure 5A, the results revealed a notable negative correlation between *AURKA* expression and the immunoinfiltration score in colon cancer tissues, suggesting that higher *AURKA* expression might be associated with reduced immune cell infiltration. In contrast, expressions of *TIMP1* and *NOX4* exhibited significant positive correlations with immune infiltration, implying that these genes could be enhancing immune cell presence in the tumor microenvironment.

Interestingly, no significant correlation was observed with *TP53* expression, indicating that the dysregulation of *TP53* might not significantly affect the immune infiltration in colon cancer, unlike other studied genes. This suggests that the abnormal expressions of *AURKA*, *TIMP1*, and *NOX4* may play more pivotal roles in modulating the immune landscape during the oncogenesis and progression of colon cancer.

#### 3.4.2 Association of core gene expression with immune cell infiltration in cancer tissues

The immune infiltration profiles of four pivotal genes *NOX4*, *TIMP1*, *AURKA*, and *TP53* were exhaustively analyzed using the TIMER (Tumor Immunity Estimation Resource) database, with a particular emphasis on COAD (colon adenocarcinoma) as the tumor type. The assessment encompassed a range of immune cells including B cells, CD8^+^ T cells, CD4^+^ T cells, macrophages, neutrophils, and dendritic cells to obtain a comprehensive understanding of the impact of each gene on the tumor microenvironment.

The analysis yielded scatter plots (Figure 5B), which depicted the relationship between the expression of the four core genes and both tumor purity and the infiltration levels of six different types of immune cells. Of particular interest is the observation that *NOX4* exhibited a negative association with B-cell immunity levels, suggesting the potential for inhibitory effects on this cell type. However, a contrasting positive correlation was observed with the infiltration of the remaining immune cells. In contrast, *TP53* demonstrated a generally positive correlation with immune cell levels across the spectrum, particularly with dendritic cells, highlighting its potential role in enhancing immune surveillance against tumor cells. *TIMP1*, while showing no significant correlation with CD4^+^ T cell levels, was positively associated with the infiltration of other immune cell types, indicating its involvement in modulating the immune environment. *AURKA*, on the other hand, demonstrated an absence of significant associations with the infiltration levels of any of the six analyzed immune cell types, suggesting a minimal direct impact on immune infiltration in the context of colon cancer. This analysis underscores the differential impact of core genes on the immune landscape of colon cancer, revealing complex interactions that could influence both the progression of the disease and the response to therapy.

#### 3.4.3 Nuclear core gene expression and immune cell analysis in colon cancer tissues

The initial step in our analysis entailed the importation of sample data to extract gene expression profiles from tumor tissues, which were subsequently mapped to GeneSymbol with high precision. Subsequent data processing utilized the R package IOBR (version 0.99.9, available at https://www.ncbi.nlm.nih.gov/pmc/-articles/PMC828378), which facilitated the conversion of data into the IOBR format suitable for in-depth immune infiltration analysis. A comprehensive reevaluation was conducted to assess the filtration levels of various immune cells, including B-cells, CD4^+^ T cells, CD8^+^ T cells, neutrophils, macrophages, and DCs (dendritic cells) in each patient’s tumor based on gene expression profiles (Li et al., 2017). This method known as the TIMER method (Tumor Infiltration Web server for integrated immune cell analysis) provided a refined insight into the immune landscape of the tumor microenvironment. Our analysis revealed the patterns of gene expression correlation with immune cell infiltration (Figure 5C). *AURKA* and *TP53* exhibited no significant correlation with the infiltration scores of the six evaluated immune cell types, suggesting their minimal direct impact on modifying the immune microenvironment in the context of the tumor types studied. In contrast, *NOX4* and *TIMP1* exhibited correlations with immune cell infiltration to varying degrees. Specifically, the expression of these genes was found to exert a more pronounced influence on the infiltration states of the six immune cells, indicating their potential roles in modulating immune responses within the tumor microenvironment.

#### 3.4.4 Detection of immune checkpoints

To explore the potential roles of *NOX4*, *TIMP1*, *AURKA*, and *TP53* genes in immune regulation and their interaction with immune checkpoint pathways, expression data for these core genes was derived from a comprehensive pan-cancer dataset, alongside data from 60 immune checkpoint pathway genes (24 inhibitory and 36 stimulatory). Each gene expression value underwent a log_2_ (x+0.001) transformation to normalize the data and facilitate subsequent analyses.

The Pearson correlation coefficients were calculated to assess the relationships between the expression of the four core genes and the marker genes associated with five types of immune pathways. The expression levels of *NOX4*, *TIMP1*, *AURKA*, and to a lesser extent *TP53*, showed significant correlations with a majority of the immune marker proteins (Figure 5D).

A noteworthy finding was the observation of a correlation between *PD-1* (*PDCD1*), a pivotal immune checkpoint protein, and all four core genes. This finding suggests a more extensive role for these genes in regulating immune checkpoint activity. This observation underscores the significance of these genes, particularly in their interaction with *PD1*, in influencing tumor suppression by affecting immune evasion mechanisms.

#### 3.4.5 Significant upregulation of core gene expression observed in clinical colon cancer tissue specimens

IHC (Immunohistochemistry) was utilized to evaluate the expression levels of select genes, including *NOX4*, *TIMP1*, *AURKA*, and *TP53* in clinical colon cancer specimens. The objective of this analysis was to provide a visual and quantitative comparison of protein expression between cancerous and normal colon tissues. As illustrated in Figure 5E, the IHC experiments revealed that the proteins corresponding to the genes *NOX4*, *TIMP1*, *AURKA*, and *TP53* exhibited significantly higher levels in colon cancer tissues compared to their expression in adjacent normal tissues.

The elevated expression of these proteins in tumor tissues underscores their potential roles in the pathogenesis of colon cancer. The differential expression patterns observed suggest that these genes may contribute to tumorigenic processes such as proliferation, invasion, and immune evasion.

### 3.5 Mechanistic pathway exploration of key genes

#### 3.5.1 PMFs inhibit ferroptosis-related protein and PD-L1 expression

**FIGURE 6:**
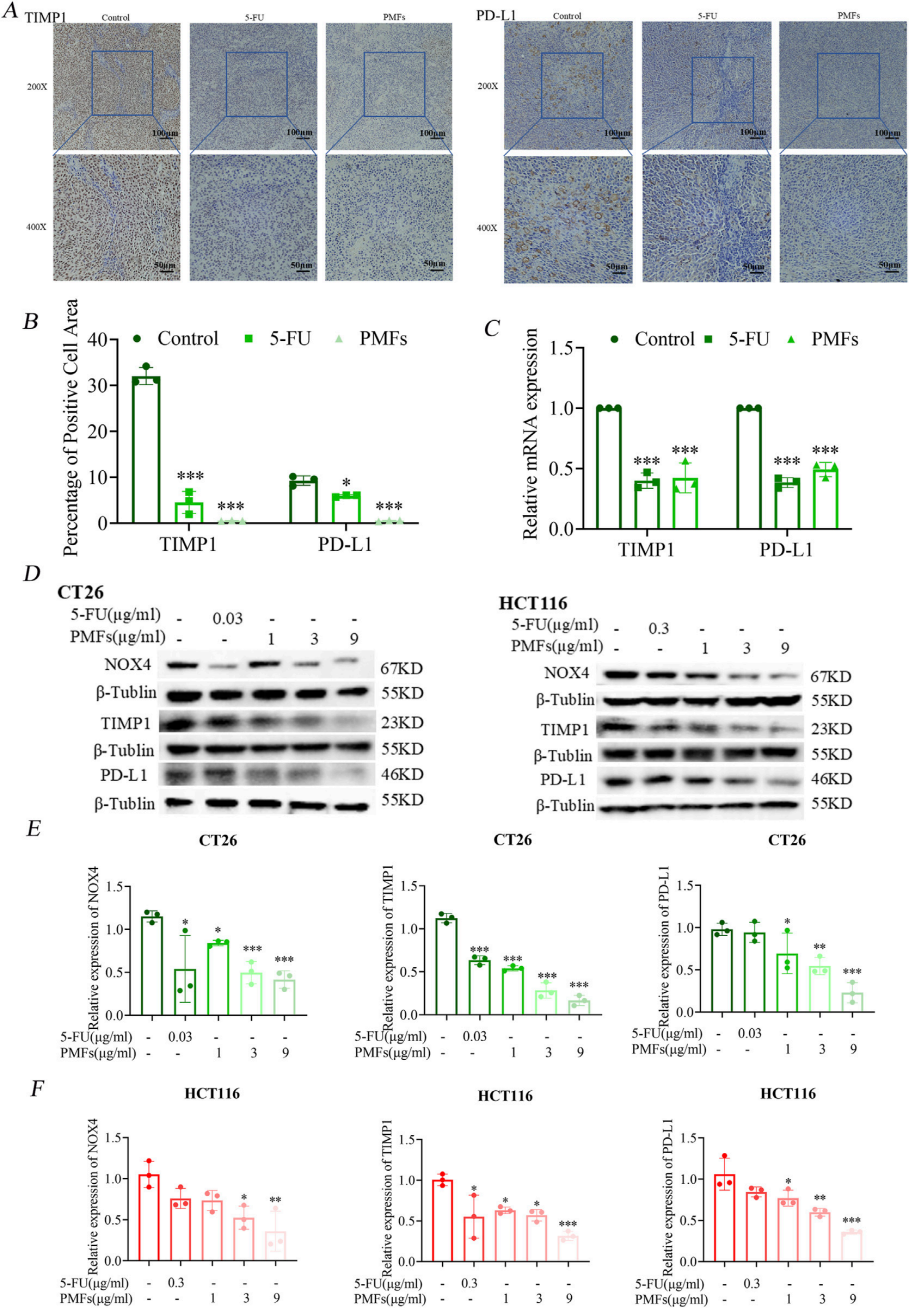
PMFs inhibit ferroptosis-related protein and *PD-L1* expression. **(A, B)** Immunohistochemical staining and statistical graphs of *TIMP1* and *PD-L1* in tumour tissue sections under 200X and 400x microscope. **(C)** Histograms of relevant mRNA expression of potential ferroptosis-related genes *TIMP1* and *PD-L1*. **(D)** The changes in protein bands of *NOX4*, *TIMP1,* and the immunocheckpoint molecule *PD-L1*, which were screened through bioinformatic immunoassay, after CT26 and HCT116 cells were treated with different concentrations of PMFs. **(E, F)** Statistical graphs of protein expression on CT26 and HCT116 treated with different concentrations of PMFs. *P < 0.05, **P < 0.01, and ***P < 0.001.

The xenograft tumor tissues constructed in the previous stage were sectioned, and then immunohistochemical analysis was performed to evaluate the expression of *TIMP1* and *PD-L1*. The results showed that the proportions of cells with positive staining for *TIMP1* and *PD-L1* in the 5-FU and PMFs groups were lower than those in the control group (Figures 6A, B).

Meanwhile, two colorectal cancer cell lines, CT26 and HCT116, were used to observe the effects of PMFs on the expression levels of key proteins. The results of Western blot analysis demonstrated that after the administration and treatment with PMFs, the expression levels of *NOX4*, *TIMP1*, and *PD-L1* were significantly downregulated (Figures 6D–F).

Notably, the downregulation of *NOX4* is contradictory to the promotion of ferroptosis. Therefore, real-time quantitative reverse transcription polymerase chain reaction (RT-PCR) was further employed to detect only the mRNA levels of *PD-L1* and *TIMP1* after treatment with PMFs. The results showed that the expressions of both were downregulated, suggesting a potential correlation between *TIMP1* and *PD-L1*, which warrants further investigation (Figure 6C). PMFs increase CD4^+^ T cell infiltration in colon cancer tissues.

**FIGURE 7:**
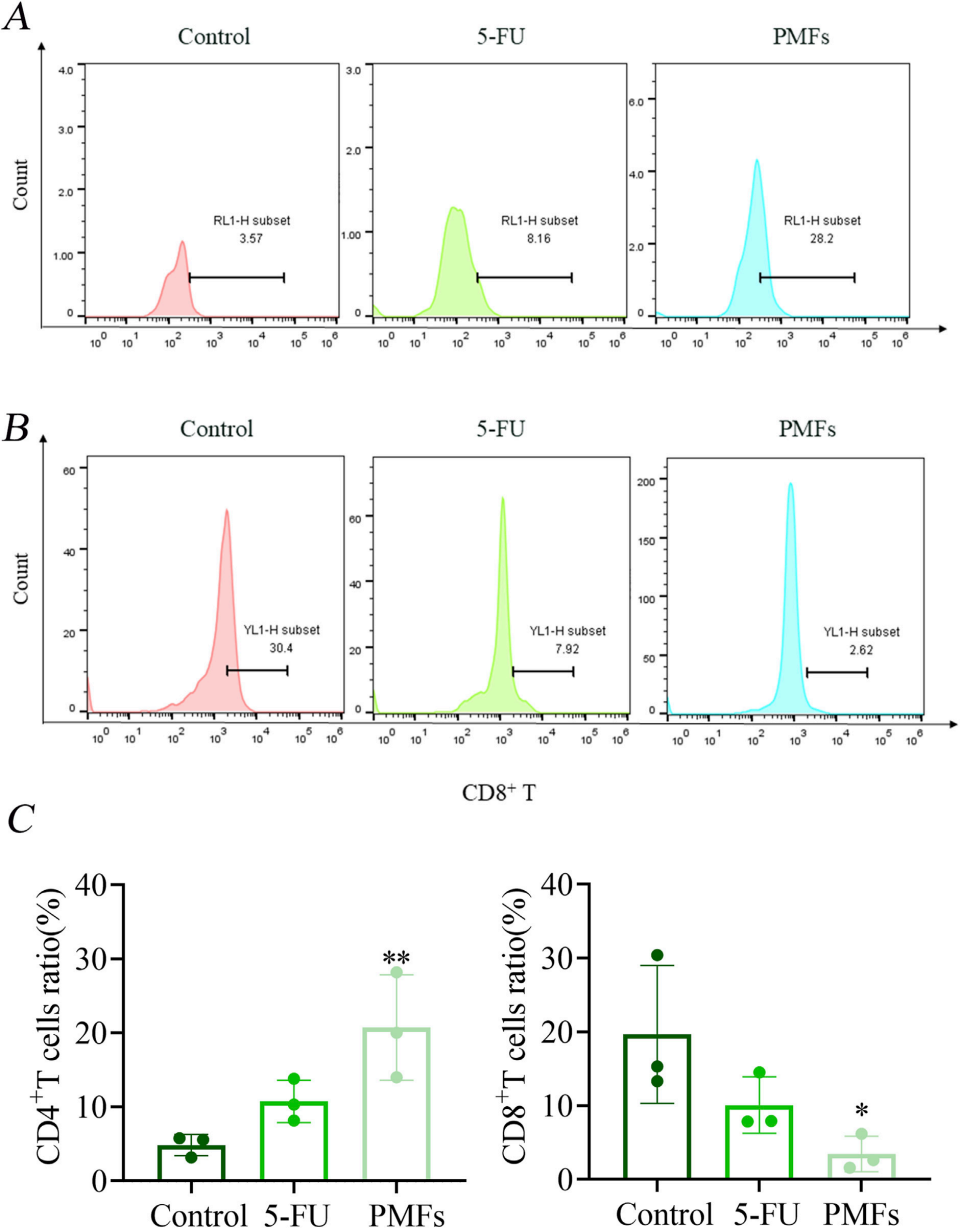
Flow Detection of Immunological Factors. **(A, B)** Detection of CD4^+^ T and CD8^+^ T in different treatment groups; **(C)** Statistical plots of percentage content of CD4^+^ T and CD8^+^ T. *P < 0.05, **P < 0.01, and ***P < 0.001.

Flow cytometry was used to assess the response of immune cell populations to PMFs treatment in a colon cancer model. In the 5-FU-treated and PMFs-intervention groups, the number of CD4^+^ T cells was significantly increased, whereas the infiltration of CD8^+^ T cells was reduced, previous studies have shown that 5-FU therapy correlates with an elevation in CD4^+^ T cells and a reduction in CD8^+^ T cells (Tian et al., 2021; Yang et al., 2023). Notably, recent advances in the field have highlighted that CD8^+^ T cells, when activated by immunotherapeutic approaches, exhibit heightened sensitivity to ferroptosis, a form of iron-dependent cell death. Additionally, CD8^+^ T cells themselves are more susceptible to ferroptosis compared to the tumor cells, which may restrict the therapeutic efficacy of ferroptosis inducers in cancer treatment (Li et al., 2024; Lin et al., 2024). This indicated that PMFs had a positive effect on CD8^+^ T cell activity and anti-tumor immune response (Figures 7A–C).

## 4 Discussion

Recent advancements in the field of immunotherapy have established it as a fundamental component in the therapeutic landscape of oncology, particularly through the ICIs (implementation of immune checkpoint inhibitors). These therapeutic modalities have demonstrated considerable promise, attributable to their capacity to reinvigorate T-cell functionality, thereby enabling the targeted eradication of tumor cells (Sanmamed and Chen, 2018). Specifically, ICIs have demonstrated increased efficacy in CRC subpopulations characterized by dMMR (defective mismatch repair) and or MSI-H (high microsatellite instability) (Lin et al., 2023). Despite these advances, the overall impact of immunotherapy remains limited in broader CRC patient populations (Ciardiello et al., 2019).

Adding to the complexity of the immune landscape in cancer, recent studies have identified ferroptosis—a form of programmed cell death dependent on iron—as a significant influencer of immune cell behavior, affecting their survival, differentiation, and effector functions (Matsushita et al., 2015; Ito et al., 2020; Muri et al., 2019). This process not only facilitates the direct elimination of tumor cells but also enhances tumor antigen presentation and the recruitment and activation of immune cells within the TME. Notably, CD8^+^ T cells activated by immunotherapy can induce lipid peroxidation specific to ferroptosis in tumor cells, which supports the recruitment and activation of other immune cells (Elliott and Ravichandran, 2016).

Recent findings indicate a symbiotic relationship between ferroptosis and immune cell efficacy. For instance, ferroptotic cells release signaling molecules that aid immune cells in recognizing and phagocytizing dying tumor cells, with lipid mediators playing a pivotal role in this process (Wen et al., 2019; Klöditz and Fadeel, 2019). This underscores the potential of combining immunotherapy with ferroptosis inducers as a potent therapeutic strategy in cancer management.

The role of the *TIMP1* has also beenexamined within this context. Elevated *TIMP1* expression in CRC has been associated with various immune cells, suggesting its involvement in the immune response to tumorigenesis (Pan et al., 2023; Xie et al., 2024; Xu et al., 2023). Studies have demonstrated that *TIMP1* is associated with the regulation of immune checkpoints and negatively correlates with patient outcomes, indicating its potential as a therapeutic target (Hu et al., 2023; Zhang et al., 2023; Qiu et al., 2021). *TIMP1* is markedly expressed in CRC tumors, and elevated *TIMP1* expression is associated with unfavorable clinical outcomes in CRC patients (Kobayashi et al., 2015; Lee et al., 2011). This finding is corroborated by our immunohistochemical and survival analyses.

Furthermore, there is emerging evidence of the co-expression of *TIMP1* with both *CXCL8* and *c-MYC*, which may influence *PD-L1* expression and thus, the immune escape mechanisms of tumor cells (Sun et al., 2018; Huang et al., 2021). Our preliminary investigations using Western blotting and quantitative PCR have begun to elucidate the complex interactions between these molecules. However, a more comprehensive understanding of the regulatory pathways involving *TIMP1*, *CXCL8*, and *PD-L1* is necessary to fully capitalize on the therapeutic potential of targeting these pathways.

In conclusion, the incorporation of ferroptosis-inducing agents into established immunotherapeutic strategies presents a promising avenue for enhancing the efficacy of cancer treatments. This approach has the potential to transform the treatment landscape for CRC by enhancing immune system engagement and overcoming resistance mechanisms. Further research is essential to explore these interactions and their implications for clinical practice, aiming to halt the progression of colon cancer more effectively.

## Data Availability

The original contributions presented in the study are included in the article/Supplementary Material, further inquiries can be directed to the corresponding authors.
