# Supplementary material for: Elucidation of the mechanism Underlying the promotion of ferroptosis and enhanced antitumor immunity by citrus polymethoxyflavones in CRC cells
Source: Front Pharmacol. 2025 Apr 11;16:1571178. doi: 10.3389/fphar.2025.1571178 (PMC12021823; doi:10.3389/fphar.2025.1571178)
Supplement: Supplementary file 1 [file Supplementaryfile1.docx]

**Supplementary Figures**


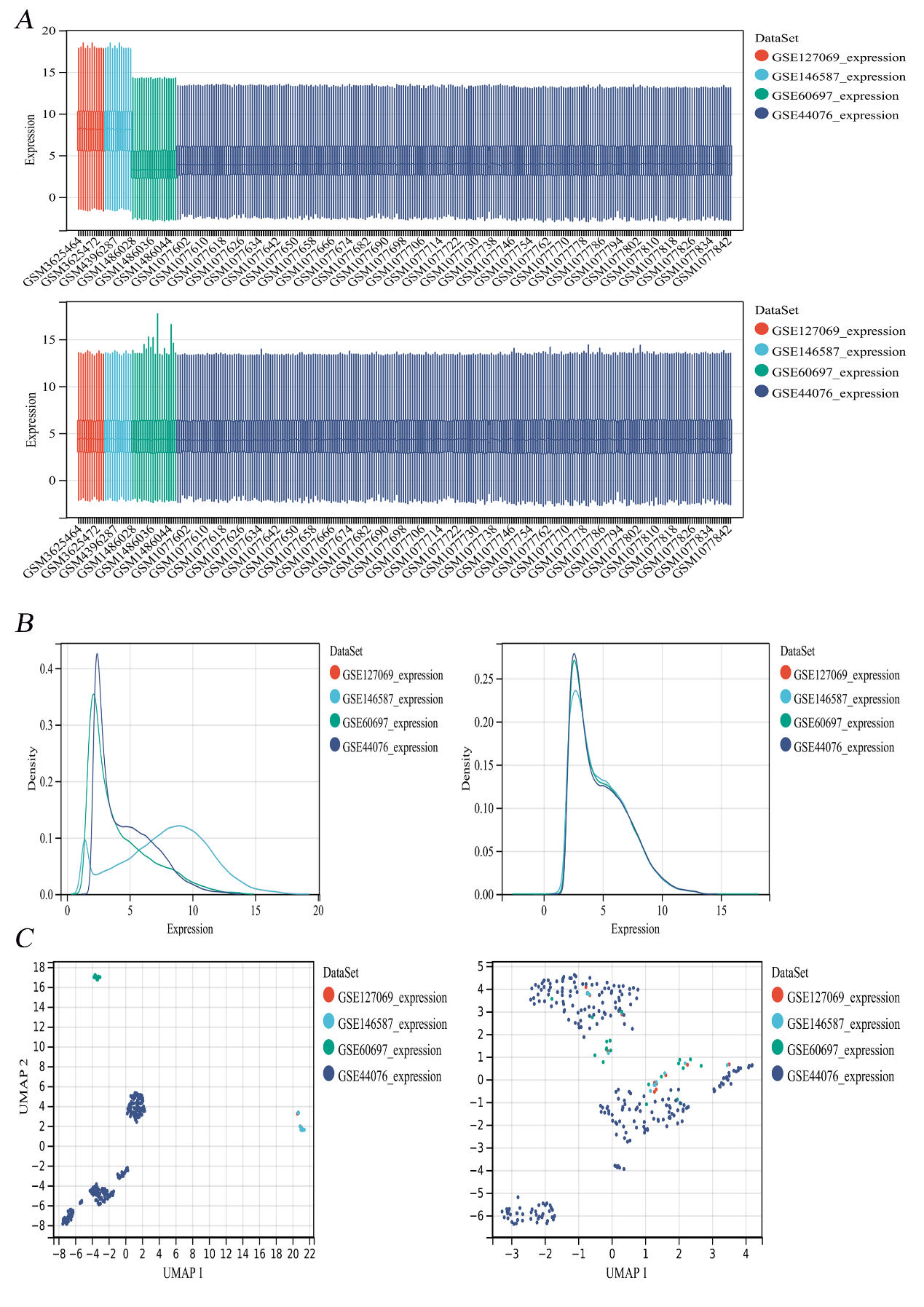


**Figure S1:(A-C)** shows binary plots (to validate the removal effect and uncover the true differences), density plots (to assess the homogeneity improvement and show the true distribution) and UAMP plots (to validate the integration and to facilitate comparative analyses of multiple data sets) before and after the removal of the batch effect for four datasets: GSE146587, GSE127069, GSE44076 and GSE60697.


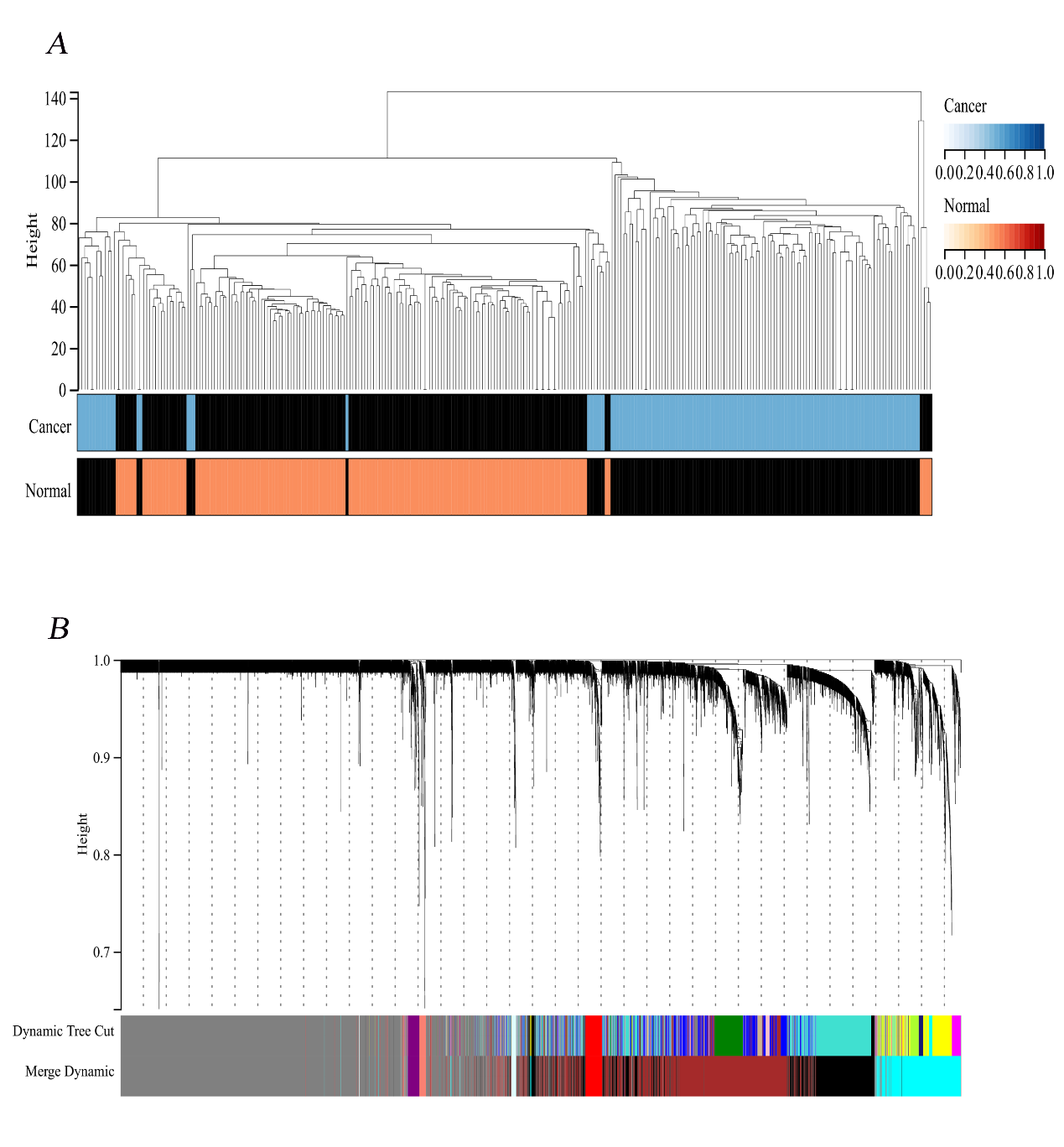


**Figure S2:** **(A-B)** shows sample clustering (discovering sample subpopulations, assessing sample quality and consistency) and gene clustering (identifying functional modules, mining inter-gene interrelationships) of the WGCNA weighted co-expression network.


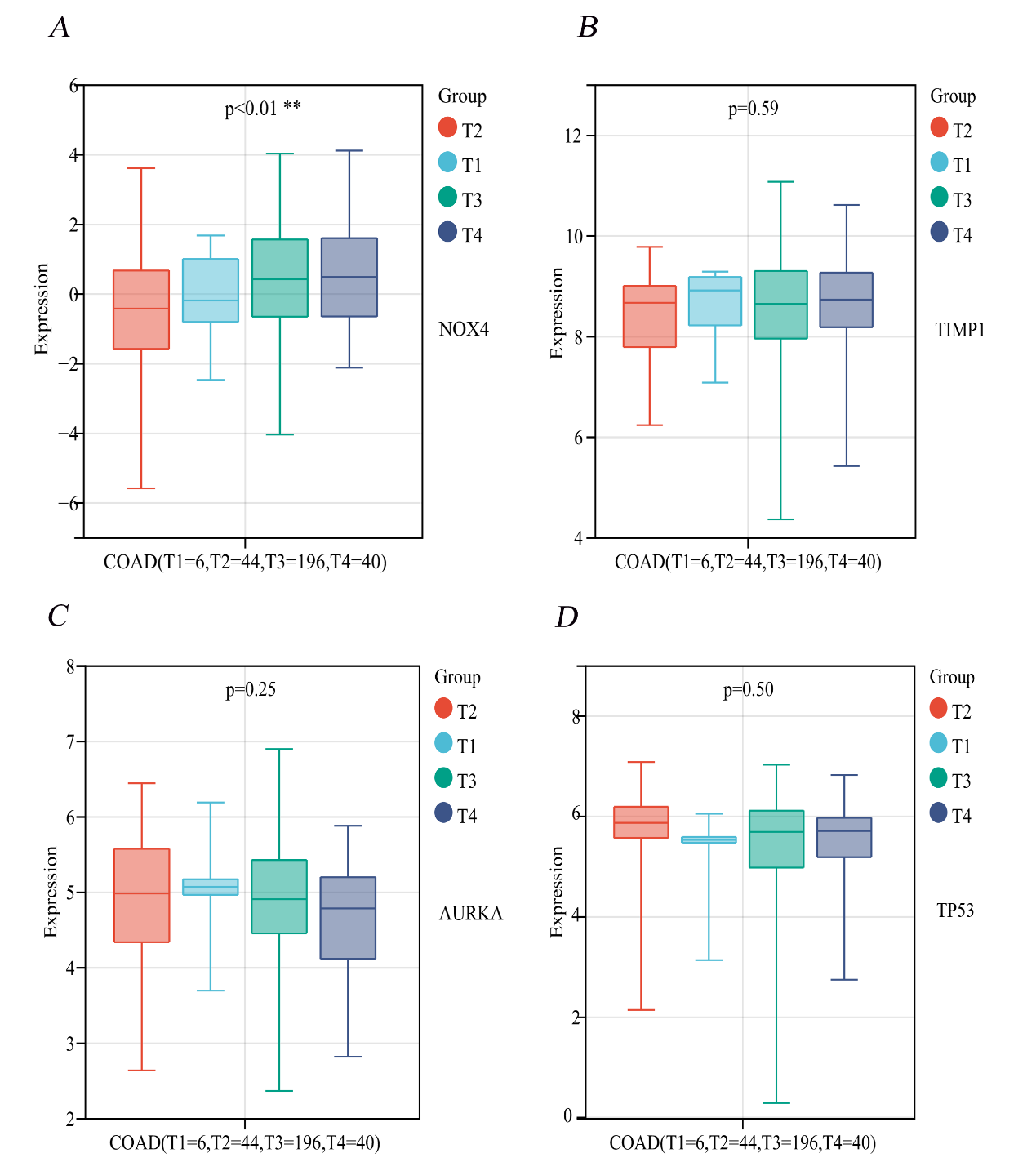


**Figure S3: (A-D)** shows the box plots of NOX4, TIMP1, AURAKA and TP53 expression and clinical staging. The relationship between gene expression differences and clinical stage was visually demonstrated to assess the stage-specific changes in gene expression, assist prognosis judgement and screen therapeutic targets.
